# A Novel Nomogram Based on Hepatic and Coagulation Function for Evaluating Outcomes of Intrahepatic Cholangiocarcinoma After Curative Hepatectomy: A Multi-Center Study of 653 Patients

**DOI:** 10.3389/fonc.2021.711061

**Published:** 2021-07-12

**Authors:** Yunshi Cai, Bohan Zhang, Jiaxin Li, Hui Li, Hailing Liu, Kunlin Xie, Chengyou Du, Hong Wu

**Affiliations:** ^1^ Department of Liver Surgery & Liver Transplantation, State Key Laboratory of Biotherapy and Cancer Center, West China Hospital, Sichuan University and Collaborative Innovation Center of Biotherapy, Chengdu, China; ^2^ Department of Hepatobiliary Surgery & Liver Transplantation, The First Affiliated Hospital of Chongqing Medical University, Chongqing, China

**Keywords:** gamma-glutamyl transpeptidase, GGT to platelet ratio, intrahepatic cholangiocarcinoma, international normalized ratio, nomogram

## Abstract

**Background and Aims:**

Hepatic and coagulation function are routine laboratory tests prior to curative hepatectomy. The prognostic value of gamma-glutamyl transpeptidase (GGT) to platelet ratio (GPR) and international normalized ratio (INR) in surgically treated patients with intrahepatic cholangiocarcinoma (ICC) remains unclear.

**Methods:**

ICC patients received curative hepatectomy in two west China centers were included. Time-dependent ROC curves were conducted to compare established indexes with prognostic value for ICC. GPR-INR score was introduced and evaluated using the Time-dependent AUC curve and Kaplan-Meier survival analysis. A novel nomogram based on the GPR-INR score was proposed; Harrell’s C-index, calibration curve and decision curve analysis were used to assess this nomogram.

**Results:**

A total of 653 patients were included. The areas under ROC curves of GPR and INR in OS and RFS were superior to other indexes. Patients with a high GPR-INR score (1,2) presented significantly decreased overall survival (OS) and recurrence-free survival (RFS); GPR-INR sore, along with several clinicopathological indexes were selected into the nomogram, the calibration curve for OS probability showed good coincidence between the nomogram and the actual surveillance. The C-index of the nomogram was 0.708 (derivation set) and 0.746 (validation set), which was more representative than the C-indexes of the GPR-INR score (0.597, 0.678). In decision curve analysis, the net benefits of the nomogram in derivation and validation set were higher than Barcelona Clinic Liver Cancer staging (BCLC) classification and American Joint Committee on Cancer (AJCC) TNM 8^th^ staging system.

**Conclusions:**

The proposed nomogram generated superior discriminative ability to established staging systems; it is profitable to applicate this nomogram in clinical practice.

## Introduction

Liver cancer remains the second leading cause of tumor-related death in Asia, America and Africa, which leads to nearly 548,400 male deaths and 233,300 female deaths annually (1). Intrahepatic cholangiocarcinoma (ICC) is one of the subtypes of cholangiocarcinoma which originates in the secondary bile ducts of the liver, which accounts for 10% to 30% of all primary liver malignancies, ranks only after hepatocellular carcinoma (2, 3). The reported global incidence of ICC increased from 0.32-0.44 to 0.85-1.18 per 100,000 population for the past half-century, the mortality rate rose synchronously from 0.1 to 1.5 per 100,000 population, highest in Asian population (1.4 per 100,000) compared with the white (0.8 per 100,000) and the black people (0.7 per 100,000) (4–7). Consistent with HCC, liver cirrhosis and HBV, HCV infections are the most relevant risk factors for ICC; other established factors including parasite infection, hepatolithiasis, primary sclerosing cholangitis, and bile-duct cyst (8, 9). ICC is associated with rapid progression, early recurrence and unsatisfying outcomes, 75% of the patients die within one year since diagnosis and the 5-year overall survival remains under 5% (4, 10). Surgical resection remains the buttress for treatment of ICC; however, survival following resection of ICC remains restrained; the median disease-specific survival time in patients received resection is 36 months (11), the 5-year overall survival rate of patients underwent R0 or R1 resection is 28% (12). Gemcitabine, fluoropyrimidine and Cisplatin-based systematic chemotherapy is beneficial to patients with recurrent, advanced or metastatic ICCs, but the median survival time was generally less than 12 months (7). Therefore, precise prognostic indexes or models are needed for evaluating the survival of surgical candidates thus improve outcomes.

Clinical and histopathological parameters including tumor size, vascular invasion, lymph node status and extrahepatic metastasis have been proved relevant with the survival of ICC patients (11, 13). These parameters are commonly used in tumor staging of the American Joint Committee on Cancer (AJCC) TNM system (14). In addition to the tumor staging system, serum markers such as carcinoembryonic antigen (CEA) and cancer antigen 19-9 (CA19-9) have been proved as independent risk factors in predicting the prognosis of ICC patients (15). Inflammatory models including neutrophil-to-lymphocyte ratio (NLR), platelet-to-lymphocyte ratio (PLR) and systemic immune-inflammation index (SII) (16) were introduced recently to predict prognosis of ICC patients; moreover, preoperative hepatic function indexes such as fibrosis-4 score (FIB-4) and albumin-bilirubin score (ALBI) also play crucial roles in predicting survival of HCC patients (17). Gamma-glutamyl transpeptidase(GGT)-to-platelet ratio (GPR), firstly defined by Lemoin et al. (18), is a more accurate marker than FIB-4 in staging HBV-related liver fibrosis; its value in predicting HCC development was further validated by Korean researchers (19). Model for end-stage liver disease (MELD) is a general standard for evaluating survival of end-stage liver disease patients and liver transplantation recipients, international normalized ratio (INR) is applied to assess coagulation function in this model (20). Furthermore, elevated INR level is associated with poor long-term outcome of HCC after hepatic resection (21). However, the prognostic value of these hepatic function parameters and coagulation indexes in ICC patients remain largely unknown.

In the present study, we compared several inflammatory, hepatic function and coagulation indexes, aimed to identify and validate the most valuable prognostic indicators for ICC patients after curative liver resection. Moreover, we hoped to establish a simple and feasible preoperative scoring system to evaluate outcomes of surgically treated ICC patients.

## Materials and Methods

### Study Population

A total of 665 surgically treated ICC patients at two west China medical centers, West China hospital of Sichuan University and the First Affiliated Hospital of Chongqing Medical University, were sequentially included. 535 patients received the operation at the West China Hospital during December 2008 and December 2017 were enrolled as derivation set to assess the parameters and establish the nomogram, 130 patients received the operation at the First Affiliated Hospital of Chongqing Medical University between May 2010 and December 2015 were included as the validation set to verify the effectiveness of the model. The inclusion criteria were as follow: (1) histologically diagnosed ICC; (2) underwent hepatectomy with curative intent firstly; exclusion criteria: (1) patients received preoperative transarterial chemoembolization (TACE), radiofrequency ablation (RFA), radiotherapy, targeting therapy, or other anti-tumor treatment; (2) insufficient hepatic functional reserve for liver resection; (3) extrahepatic metastasis; (4) positive surgical margin; (5) tumor rupture; (6) patients without complete clinical data and follow-up information. Written informed consents were obtained from all participants or their relatives. This study was approved by the ethics committee of West China Hospital of Sichuan University and the First Affiliated Hospital of Chongqing Medical University, in line with the guidelines of the 1975 Declaration of Helsinki.

### Data Collection and Follow-Up

All the clinical and histopathological information were obtained from the hospital electronic medical records. Preoperative data were collected as follows: neutrophil, lymphocyte and platelet counts; bilirubin, albumin and GGT levels; alanine aminotransferase (ALT), aspartate aminotransferase (AST) and INR levels; HBV and HCV loads. Moreover, the serum tumor markers including alpha-fetoprotein (AFP), carcino-embryonic antigen (CEA) and carbohydrate antigen 19-9 (CA19-9) levels were collected. the GPR was defined as GGT level (U/L) divided by platelet counts (10^9^/L); platelet-to-lymphocyte ratio (PLR) was calculated from platelet counts divided by lymphocyte counts; systemic immune-inflammation index (SII) was calculated from (*neutrophil*(10^9^/L) × *Platelet*(10^9^/L))/*Lymphocyte*(10^9^/L00; fibrosis-4 index (FIB-4) was calculated from $\left(ALT(U/L)\times age(year))/(platelet(10^{9}/L)\times \sqrt{AST(U/L)}\right)$; ALBI was calculated by the following formula: (*log*_10_ *bilirubin*(*mol*/*L*) × 0.66) – (*albumin*(*g*/*L*) × 0.085). X-tile software (22) was used to determine the cut-off values of these indexes and patients were stratified according to the cut-offs. Clinical and histopathological features including ascites, liver cirrhosis, numbers of nodules, diameter of tumor, differentiation, hepatolithiasis, lymph node status, microvascular invasion (MVI), macrovascular invasion, perineural invasion and extrahepatic metastasis were also obtained. MVI was defined as the presence of tumor cells in vessels or in vascular space lined by the epithelial cells under the microscope; macrovascular invasion was defined as radiologically or macroscopically identified large vessel invasion. Para-aortic lymph node invasion was considered as extrahepatic metastasis. All included patients were stratified according to the 8^th^ edition of AJCC staging manual and the Barcelona Clinic Liver Cancer (BCLC) staging classification (14, 23). Postoperative follow-up was conducted per month within 1 year, and then every 3 months within two years, then every 6 months thenceforth, serum tumor markers and contrast-enhanced ultrasonography or CT scan were used to monitor the recurrence of tumor. For patients who had difficulties going back to the hospital for routine examination due to distance or traffic issues, we recommend them to exam in local medical institutions and re-check the findings through telephone and Internet follow-up surveys. At the end of follow-up in December 2017, 8 patients (5 patients in West China Hospital and 3 in the First Affiliated Hospital of Chongqing Medical University) lost to follow-up. 4 patients in the First Affiliated Hospital of Chongqing medical university were withdrawn due to incomplete medical records (pathology report or preoperative laboratory indexes).

### Statistical Analysis

The software of EmpowerStats (http://www.empowerstats.com) and R (https://www.r-project.org, v3.6.3) were used for statistical analysis, data was presented as mean ± standard deviation (SD) or proportion. Comparison of categorical and continuous variables between groups were performed with Student’s *t*-test and Pearson’s *x^2^* test, respectively. Non-parametric Mann-Whitney U test was used to analysis the data with abnormal distribution. The ideal cut-off values of GPR, INR, PLR, SII, FIB-4 and ALBI were determined by using X-tile software (http://medicine.yale.edu/lab/rimm/research/software). The discriminative ability of the indexes was assessed by the area under receiver operating characteristic curves (AUROC) or time-dependent AUROC *via* the “survivalROC” package in R. Kaplan-Meier curves were depicted according to the cut-off values of GPR, INR and GPR-INR score, and their differences between groups were determined by comparing the cumulative survival of included ICC patients using the log-rank test. Cox proportional hazards regression model was used to identify potential prognostic factors for overall survival (OS), clinicopathological indexes with *P*<0.2 in the univariate model were incorporated into the multivariate Cox regression model, and the nomogram was generated accordingly. Harrell’s concordance index (c-index) and calibration curves were applied to evaluate the availability of the nomogram; comparisons between the nomogram and other staging systems were achieved by using decision curve analysis (DCA) *via* the “rmda” package in R. *P*<0.05 was considered statistically significant.

## Results

### Patients Characteristics at Baseline

530 patients [256 (48.3%) male, mean (SD) age, 57.2 (10.7) years] in West China Hospital and 123 patients [71 (57.7%) male, mean (SD) age, 58.2 (11.1) years] in the First Affiliated Hospital of Chongqing Medical University were finally recruited into the derivation set and validation set, respectively. Liver cirrhosis was detected in 148 (27.9%) patients in the derivation set and 16 (13%) patients in the validation set; all patients were stratified into Child-Pugh grade A, in derivation set, 451 (85.1%) patients were 5 points and 79 (14.9%) were 6 points; in the validation set, 93 (75.6%) were 5 points and 30 (24.4%) were 6 points. Nearly 30% of the patients were with multiple tumors in both cohorts, and tumor diameter on average was greater than 5 cm (5.9 ± 2.7 cm in derivation set and 6.2 ± 1.4 cm in validation set). More than half of the patients in both sets were with poor tumor differentiation (63.4% in derivation set and 73.2% in validation set). 53 (10%) patients in derivation set and 15 (12.1%) patients in the validation set were with microvascular invasion, 138 (26%) patients in derivation set and 30 (24.4%) patients in the validation set were with elevated serum CA19-9 level (≥22U/ml); additionally, 88 (16.6%) patients in derivation set and 23 (18.7%) patients in validation set suffered from hepatolithiasis. Patients’ characteristics at baseline were summarized in **Table 1**.

**Table 1:** Baseline characteristics of patients.

Variables	Deveriation set (n=530)	Validation set (n=123)
Age, mean ± SD	57.2±10.7	58.2±11.1
Gender, n (%)		
Male	256(48.3%)	71(57.7%)
Female	274(51.7%)	52(42.3%)
Cirrhosis, n (%)	148(27.9%)	16(13.0%)
Ascite, n (%)	50(9.4%)	60(48.8%)
Child score, n (%)		
5	451(85.1%)	93(75.6%)
6	79(14.9%)	30(24.4%)
Multiple tumors, n (%)	157(29.6%)	36(29.3%)
Tumor size (cm), mean ± SD	5.9±2.7	6.2±1.4
Poor tumor differentiation, n (%)	336(63.4%)	90(73.2%)
Hepatolithiasis, n (%)	88(16.6%)	23(18.7%)
Microvascular invasion, n (%)	53(10.0%)	15(12.1%)
Macrovascular invasion, n (%)	123(23.2%)	25(20.3%)
Lymph node metastasis, n (%)	129(24.3%)	35(28.5%)
Biliary invasion, n (%)	53(10.0%)	63(51.2%)
Perineural invasion, n (%)	77(14.5%)	29(23.6%)
Liver capsule invasion	325(61.3%)	65(52.8%)
CA19-9, n (%)		
≥22U/ml	138(26.0%)	30(24.4%)
<22U/ml	346(65.3%)	93(75.6%)
Not available	46(8.7%)	0(0%)
HBsAg (positive),n(%)	153(28.9%)	22(17.9%)
HCV, n (%)	3(0.6%)	0(0%)
TNM stage, n (%)		
I-II	173(32.6%)	45(36.6%)
III-IV	357(67.4%)	78(63.4%)
BCLC stage		
0-A	268(50.6%)	47(38.2%)
B-C	262(49.4%)	76(61.8%)
INR grade		
<1.1	457(86.2%)	80(65.0%)
≥1.1	73(13.8%)	43(35.0%)
GPR grade		
<0.7	354(66.8%)	55(44.7%)
≥0.7	176(33.2%)	68(55.3%)
Overall survival, month, mean ± SD	24.9±21.4	26.2±20.8

CA19-9, cancer antigen 19-9; HBsAg, hepatitis B surface antigen; HCV, hepatitis C virus; BCLC stage, Barcelona Clinic Liver Cancer stage; INR, international normalized ratio; GPR, gamma-glutamyl transpeptidase to platelet ratio.

### GPR and INR Were Related to Increased Risk of Prognosis

The preoperative inflammatory, hepatic function and coagulation function indexes including GPR, INR, PLR, SII, FIB-4 and ALBI were calculated based on the patients’ data of derivation set, X-tile software was adopted to confirm the optimal cut-off values of these indexes (**Supplemental Figure 1**) with minimal *P*-value from log-rank *x^2^* test (GPR: 0.7, INR: 1.1, PLR: 104.4, SII: 683, ALBI: -0.8). The discriminative capability of these indexes to 1-,3- and 5-year OS and RFS were compared by using ROC; the AUC of GPR, SII and INR were superior to that of PLR, FIB-4 and ALBI (**Supplemental Figure 2**). Considering that both the values of GPR and SII were impacted by the platelet counts, the GPR and INR were selected. Patients were stratified into four groups according to the cut-off values of GPR and INR (group A: GPR<0.7, group B: GPR≥0.7; group C: INR<1.1, group D: INR≥1.1) with the strongest discriminative ability, the correlation between baseline characteristics with GPR and INR of the patients in derivation seta was demonstrated in **Table 2**. There were 354 patients in group A and 176 patients in group B; compared to group A, group B presented with the following distinctions: more male patients and fewer female patients (*P*=0.01), higher prevalence of macrovascular invasion (*P*<0.001), biliary invasion (*P*<0.001), higher Child-Pugh score (*P*=0.002), lower prevalence of liver capsule invasion (*P*<0.001), lower CA19-9 level (*P*=0.027), lower rate of the presence of positive HBsAg (*P*=0.029). Based on the cut-off of INR, 457 patients were classified into Group C and 73 in Group D; in comparison to Group C, Group D presented with: higher rate of male patients (*P*=0.003), higher rate of the presence of liver cirrhosis (*P*<0.01), higher rate of positive HBsAg (*P*<0.001) and Hepatitis C antibody (*P*=0.008), lower prevalence of liver capsule invasion (*P*=0.044), lower Child-Pugh score (*P*=0.001). There was no significant difference in other characteristics among these groups. The basic features of patients in the validation set were shown in **Supplemental Table 1**. Kaplan-Meier analysis suggested that the patients with GPR<0.7 had better OS and RFS than those with GPR≥0.7 (median OS: 25.8 months *vs*. 23.1 months), in consistence with GPR, the OS and RFS of the patients with INR<1.1 also superior to the patients with higher INR (median OS: 25.4 months *vs*. 21.3 months) (**Supplemental Figure 3**).

**Table 2:** Univariate and multivariate analyses to determine independent predictors of overall survival.

Variables	Univariate analysis			Multivariate analysis		
	HR	95%CI	P value	HR	95%CI	P value
Gender, Female/Male	0.821	0.658-1.023	0.078			
Age	0.995	0.987-1.008	0.708	0.833	0.643-1.078	0.165
Cirrhosis	1.274	1.002-1.619	0.047	1.511	1.145-1.993	0.003
Hepatolithiasis	1.323	1.005-1.751	0.046	1.344	0.976-1.851	0.07
Tumor number,Multiple/single	1.712	1.359-2.156	<0.0001	1.587	1.074-2.346	0.02
Tumor size,≥5/<5(cm)	1.212	0.969-1.517	0.092	1.237	0.941-1.626	0.126
Tumor differentiation, Undifferentiation-Poor/Moderate-Well	2.053	1.555-2.712	<0.0001	1.881	1.396-2.535	<0.0001
Microvascular invasion	1.719	1.236-2.391	0.001	1.009	0.696-1.465	0.958
Macrovascular invasion	1.181	0.914-1.526	0.203			
Lymph node metastasis	2.353	1.856-2.981	<0.0001	1.775	1.326-2.377	0.0001
Liver capsule invasion	1.078	0.858-1.352	0.517			
Perineural invasion	1.564	1.161-2.109	0.003	1.5466	1.088-2.197	0.015
GPR-INR score						
0	Reference			Reference		
1	1.806	1.395-2.341	<0.0001	1.436	1.059-1.946	0.019
2	3.233	2.226-4.696	<0.0001	2.794	1.833-4.258	<0.0001
CA199 grade ≥22/<22(U/ml)	2.166	1.621-2.895	<0.0001	2.018	1.471-2.769	<0.0001
HBV	1.131	0.889-1.437	0.317			
HCV	1.865	0.597-5.828	0.283			
TNM stage,III-IV/I-II	1.397	1.075-1.815	0.012	1.045	0.711-1.535	0.821
BCLC stage,B-C/0-A	1.608	1.276-2.028	<0.0001	1.201	0.874-1.649	0.256

CA19-9, cancer antigen 19-9; HBV, hepatitis B virus; HCV, hepatitis C virus; BCLC stage, Barcelona Clinic Liver Cancer stage; INR, international normalized ratio; GPR, gamma-glutamyl transpeptidase to platelet ratio.

### The Proposal of GPR-INR Score

**Figure 1:**
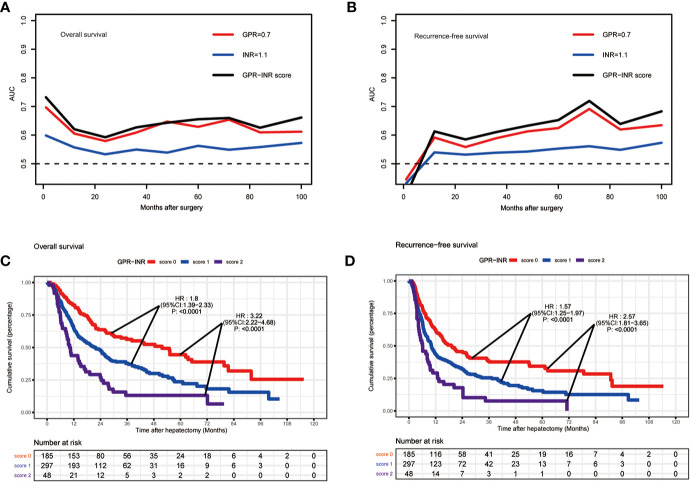
The predictive ability of GPR-INR score for OS and RFS in the derivation set. **(A, B)** The time-dependent AUC curves of GPR, INR and GPR-INR score, the AUROC of the GPR-INR score was higher than that of the GPR and the INR for RFS and OS prediction. **(C, D)** Kaplan–Meier curves of the patients in the derivation set for OS and RFS, GPR-INR score 1 had better RFS and OS than GPR-INR score 2, worse RFS and OS than GPR-INR score 0.

**Figure 2:**
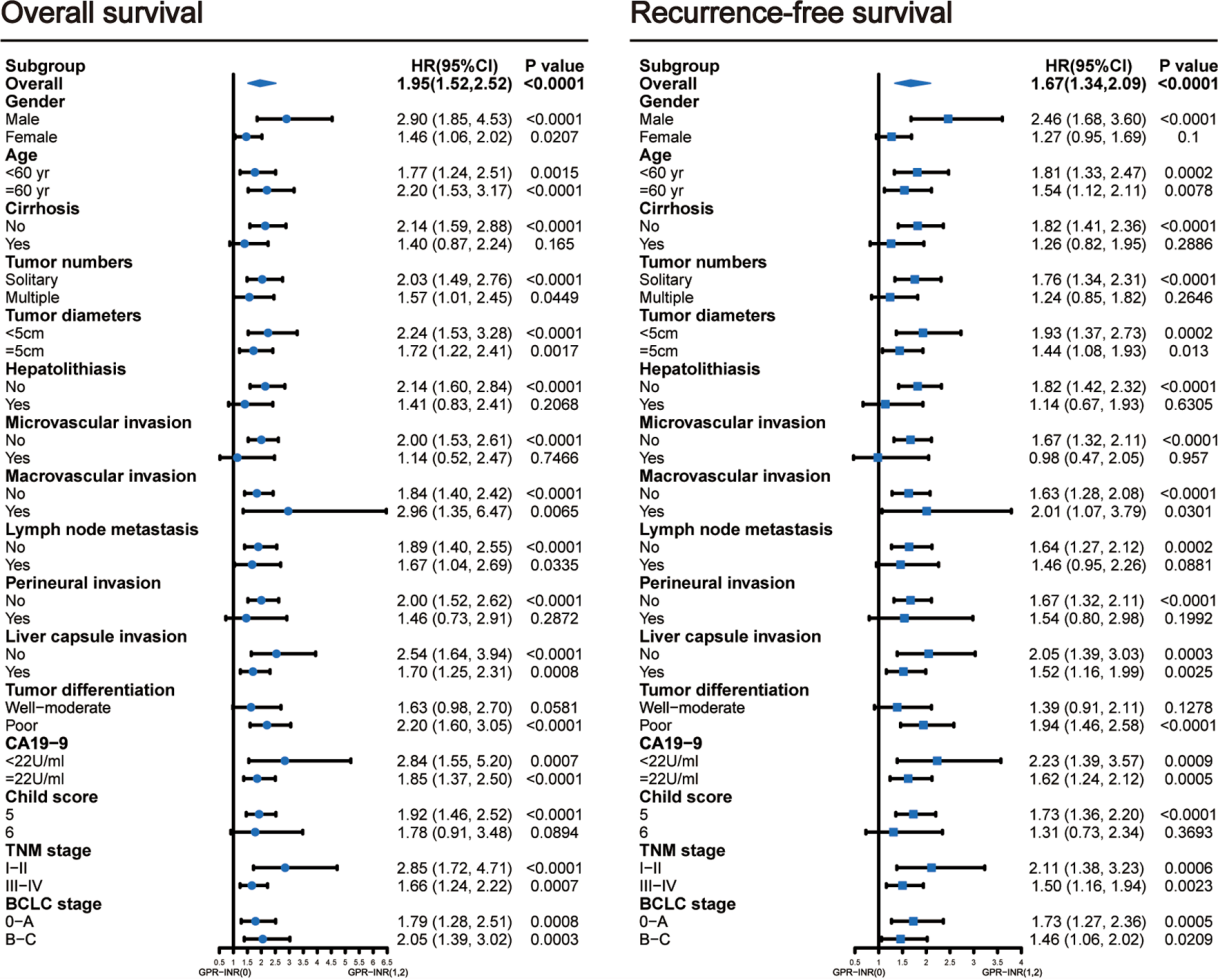
Subgroup analyses using univariable Cox regression to assess the discrimination ability of the GPR-INR score for overall survival and recurrence-free survival in patients with different clinical characteristics. CI, confidence interval.

1 point was allocated to GPR≥0.7 or INR≥1.1; therefore, the GPR-INR score composed of 0, 1 or 2 points was generated in the derivation set. Patients were stratified into three groups according to the GPR-INR score, the patients with GPR-INR score 1 had better 1-,3-,5-year OS and RFS rates than those with GPR-INR score 2 (OS: 64.9%, 20.8%, 5.4% vs. 43.7%, 10.4%, 4.2%; RFS: 41.4%, 14.1%, 4.4% *vs*. 29.2%, 6.3%, 2.1%), but worse than those with GPR-INR score 0 (OS: 64.9%, 20.8%, 5.4% *vs*. 82.7%, 30.2%, 12.9%; RFS: 41.4%, 14.1%, 4.4% vs. 62.7%, 22.1%, 10.3%) (**Figures 1C, D**). Moreover, we found that GPR-INR score surpasses GPR or INR in predicting OS and RFS through the result of time-dependent AUROC analysis (**Figures 1A, B**). To further estimate the clinical efficacy of the GPR-INR score in the prediction of OS and RFS, subgroup analysis using univariate Cox regression was applied based on gender (male or female), age (<60 or ≥60 years), tumor numbers (solitary or multiple), tumor diameters (<5 or ≥5 cm), with or without liver cirrhosis, hepatolithiasis, microvascular invasion, macrovascular invasion, lymph node metastasis, perineural and liver capsule invasion, tumor differentiation (well-moderate or poor), CA19-9 level (<22 or ≥22 U/ml), Child-Pugh score (5 or 6), TNM stage (I-II or III-IV) and BCLC classification (0-A or B-C). Although it failed to attain statistical significance in several subgroups (patients with cirrhosis, hepatolithiasis, MVI, perineural invasion etc.), the tendency of poor OS and RFS in patients with high GPR-INR score was accordant (**Figure 2**).

### Development and Validation of GPR-INR Nomogram for OS

**Figure 3:**
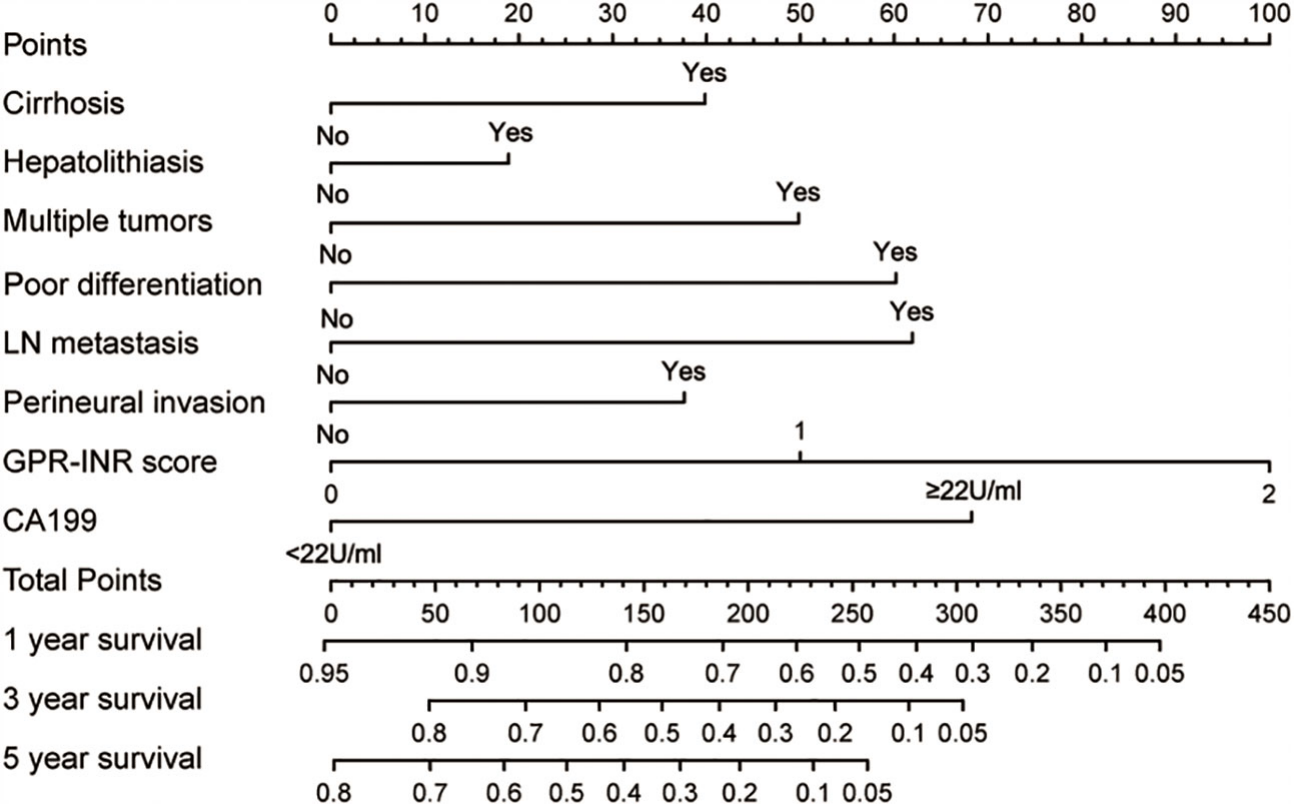
The GPR-INR nomogram. (To use the nomogram, the patient’s value was located on each variable axis, and a line was drawn to determine the individual points. The sum of these points was located on the total points axis, and a line was drawn downward to determine the likelihood of 1-, 3- or 5-year OS). CA-199, preoperative serum CA 19-9 level; LN metastasis, regional lymph node metastasis.

**Figure 4:**
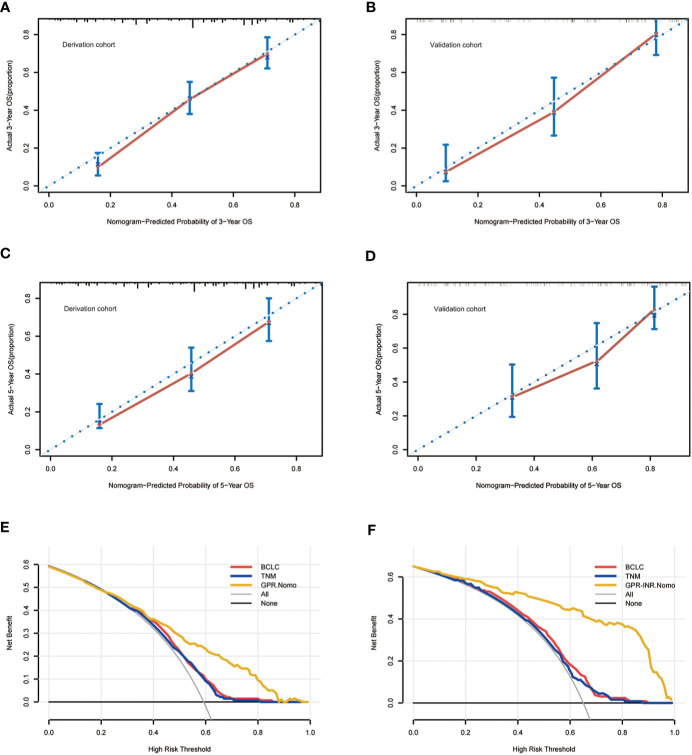
The calibration curves for predicting the OS of the patients at 3 years in the derivation set **(A)** and validation set **(B)** and at 5 years in the derivation set **(C)** and validation set **(D)**. The decision curve analysis for the BCLC staging system, the AJCC 8^th^ TNM system and the GPR-INR nomogram in the derivation set **(E)** and the validation set **(F)**.

Univariate and multivariate Cox proportional hazards regression models were used to identify independent prognostic factors for overall survival; multivariate analysis exhibited that liver cirrhosis, hepatolithiasis, multiple tumors, tumor with poor differentiation, lymph node metastasis, perineural invasion, CA19-9 and GPR-INR score were independent risk factors for overall survival. The prognostic nomogram that combined all independent risk factors for OS was developed on the basis of the patients of derivation set (**Figure 3**). C-index was compared between GPR-INR and the prognostic nomogram in the prediction of overall survival; the results suggested that the prognostic nomogram was more accurate in the evaluation of OS than the GPR-INR score (**Table 3**). The calibration curves for the probability of 3- and 5-year overall survival in derivation and validation sets were plotted, the results demonstrated an optimal accordance between the nomogram prediction and actual observation in both sets (**Figures 4A–D**). In addition, the GPR-INR nomogram was compared with two conventional systems in staging liver cancer, the BCLC and the AJCC 8^th^ edition staging systems, by using decision curve analysis. As shown in **Figures 4E, F**, the GPR-INR nomogram displayed superior net benefits within a wider range of threshold probability to the other two systems in predicting overall survival for the patients of both derivation and validation sets. The results suggested that the GPR-INR nomogram was a beneficial predictive model in predicting postoperative long-term outcomes for surgically treated ICC patients.

**Table 3:** Comparisons of the values of two models in predicting prognosis of overall survivals among the patients in derivation and validation cohort.

Models	C-index
Derivation cohort	
GPR-INR score	0.597
GPR-INR nomogram	0.708
Validation corhort	
GPR-INR score	0.678
GPR-INR nomogram	0.746

INR, international normalized ratio; GPR, gamma-glutamyl transpeptidase to platelet ratio; C-index, Harrell’s concordance-index.

## Discussion

The present study evaluated the potential effectiveness of preoperative inflammatory, hepatic and coagulation indexes in the prediction of outcomes for ICC patients following surgical resection; GPR and INR showed the greatest prognostic value among several indexes and were integrated into GPR-INR score for the first time. Moreover, a nomogram based on the GPR-INR score and other clinicopathological features identified by multivariable analysis was built and validated in derivation and validation sets. The result of this study suggested that the GPR-INR score was an independent predictor for OS and RFS in patients with different clinical characteristics. Furthermore, with greater predictive power than BCLC and AJCC 8^th^ staging systems, the GPR-INR nomogram was an optimal scoring system in the prediction of long-term outcomes for ICC patients undergoing curative hepatectomy.

There is accumulating evidence indicating that preoperative serum biomarkers play a crucial role in predicting the prognosis of liver cancer patients. The lymphocyte-based indexes including PLR, NLR, LMR and SII have been introduced as potential prognostic markers for patients with HCC (16, 24) and ICC (25) following curative liver resection. Liver function indexes including bilirubin, albumin, alkaline phosphatase (ALP), gamma-glutamyl transpeptidase (GGT), aspartate aminotransferase (AST) and alanine aminotransferase (ALT), are essential serum markers to determine the hepatic reserve function and feasibility of liver resection, on the basis of these indexes, several scoring systems including ALBI, FIB-4, AGR, AAPR and APRI, have been validated as efficient prognostic indicators for liver cancer patients (17, 26, 27). Several studies have reported that GPR demonstrated prognostic value in HCC (19, 28); however, the predictive capability of GPR for the survival of surgically treated ICC patients is unknown. As a critical indicator of coagulation function, although INR has been proposed as a promising prognostic marker in patients with HCC and biliary tract cancer (21, 29), its predictive effect in patients with ICC has not been studied separately.

Since the first prognostic nomogram for ICC was developed by Shen et al. (3), various nomograms based on different parameters have proliferated. Baseline characteristics and histopathological features were integrated by Omar et al. to build a model in predicting long-term outcomes for ICC patients following hepatectomy; the C-index for this model was 0.692 (30). Liang et al. proposed a nomogram based on radiological findings and TNM stage to predict the early recurrence of ICC (31). Tumor-related features including tumor stroma, tumor number, lymph node metastasis and MVI were combined by Jing et al. (32), with a C-index of 0.745. Xing et al. put forward a nomogram on the basis of ALBI grade to predict OS for patients with recurrent ICC (33). However, the nomogram containing preoperative hepatic and coagulation function for ICC has not yet been established.

In the current study, ROC analysis of 1-, 3-, 5-year postoperative outcome revealed that the GPR, INR and SII were superior predictive markers to predict OS and RFS in patients with ICC following surgical resection. Their predictive ability was greater than PLR, FIB-4 and ALBI, inconsistent with the tendency of the previous study in HCC patients (19). X-tile analysis determined the optimal cut-off value for the GPR and the INR. Survival analysis demonstrated that patients with a high GPR (≥0.7) and INR (≥1.1) had worse OS and RFS than those with a low GPR and INR (**Supplemental Figure 3**). High GPR and INR were more likely to present in ICC patients with liver cirrhosis, vascular and liver capsule invasion, indicating that the preoperative GPR and INR were related to the basic liver condition and tumor progression. These findings demonstrated that GPR and INR were both valuable preoperative prognostic markers for ICC patients following hepatectomy.

In order to further improve the prognostic power, the GPR and INR were integrated into the GPR-INR score; time-dependent analysis suggested that the discriminative power of the GPR-INR score was consistently greater than that of the GPR or INR in predicting long-term outcomes for surgically treated ICC patients. Patients were stratified into 3 groups according to the GPR-INR score; the ICC patients with higher GPR-INR score had worse OS and RFS in Kaplan-Meier survival analysis. In addition, the GPR-INR score remained a risk factor for OS and RFS in various subgroups.

In comparison with the GPR-INR score, the prognostic markers evaluated in this study, including SII, FIB-4, ALBI and PLR, failed to derive independent prognostic value in multivariate analysis. A prior study showed that a high preoperative PLR was an independent risk factor for OS and RFS (34); in this study, PLR was also associated with OS in univariate analysis but lost its predictive power in multivariate study, the results were inconsistent with the research from Japan (25). Similarly, the ALBI and SII showed no independent predictive utility in OS and the FIB-4 demonstrated no correlation with OS in both univariate and multivariate analysis in this study, maybe because of their common relationship with the platelet counts and weaker prognostic value than GPR-INR score. On that account, we conclude that compared with these established predictors, the GPR-INR score could more accurately predict the prognosis of ICC patients following liver resection.

For the purpose of building a more accurate prediction model, clinicopathological characteristics of ICC patients were included in the evaluation. Liver cirrhosis, hepatolithiasis, multiple tumors, poor tumor differentiation, lymph node metastasis, perineural invasion, CA19-9 and GPR-INR score were identified as risk factors for OS in univariate analysis. Multivariate analysis also identified the GPR-INR score as an independent risk factor for OS, along with CA19-9, liver cirrhosis and four histopathological factors (multiple tumors, poor tumor differentiation, lymph node metastasis, perineural invasion). These factors were further integrated into the GPR-INR nomogram, the predicted 3- and 5-year OS were in good agreements with the actual OS. In comparison with the single use of the GPR-INR score, the C-index of the GPR-INR nomogram demonstrated greater stratifying ability. Importantly, the BCLC and the AJCC TNM systems are the most commonly used staging systems worldwide (35, 36); the decision curve analysis indicated that the GPR-INR nomogram had superior clinical usefulness when compared with the two conventional systems.

There are several shortcomings that warrant consideration in this study. Firstly, although this was a multi-center study, both centers were located in the western region of China; due to the high prevalence of hepatolithiasis in this region, the accuracy of the GPR-INR nomogram might be limited by the etiology and retrospective design, further prospective international multi-institutional studies were needed to certify our initial findings. Secondly, few patients admitted for obstructive jaundice received preoperative percutaneous transhepaticcholangial drainage (PTCD) due to safety concerns; thus the GGT and bilirubin levels might be fluctuant. Third, a small number of patients received retreatment in other medical institutions after tumor recurrence; consequently, this study failed to assess the influence of postoperative treatment for recurrent ICC, which might alter the OS. In addition, the underlying mechanisms for the impact of the coagulation and hepatic functions on the survival of ICC patients were not analyzed in the present study; basic researches are required using *in vivo* and *in vitro* experiments to explore the concrete mechanisms.

## Conclusions

A preoperative GPR-INR score was a validated independent prognostic marker for surgically treated ICC patients, superior to the well-established inflammatory and liver functional indexes. Moreover, the GPR-INR nomogram represented a more reliable model than the AJCC 8^th^ and BCLC systems. With its low cost, objectivity and accessibility, it was profitable to consider the GPR-INR nomogram as a substitute model in risk stratification for ICC patients after curative hepatic resections.

## Data Availability Statement

The original contributions presented in the study are included in the article/**Supplementary Material**. Further inquiries can be directed to the corresponding authors.

## Ethics Statement

This study complied with Helsinki declaration of 1975. The ethics committee of West China Hospital of Sichuan University and the First Affiliated Hospital of Chongqing Medical University approved the protocols.

## Author Contributions

YC, HW, and CD: conceptualization. YC and BZ: data curation. YC, HL, and HlL: formal analysis. CD and HW: supervision. YC, BZ, and KX: writing—original draft. All authors contributed to the article and approved the submitted version.

## Funding

This work was supported by grants from the National Key Technologies R&D Program (2018YFC1106800), the Natural Science Foundation of China (81972747, 81872004, 81800564, 81770615, 81700555 and 81672882), the Science and Technology Support Program of Sichuan Province (2019YFQ0001, 2018SZ0115, 2017SZ0003), the Science and Technology Program of Tibet Autonomous Region (XZ201801-GB-02) and the 1.3.5 project for disciplines of excellence, West China Hospital, Sichuan University (ZYJC18008), 2021 Sichuan Science and Technology Plan Project “International cooperation in science and technology innovation/technological innovation cooperation in Hong Kong, Macao and Taiwan”, Artificial intelligence assisted precise diagnosis and minimally invasive treatment of liver tumor diseases (2021YFH0095).

## Conflict of Interest

The authors declare that the research was conducted in the absence of any commercial or financial relationships that could be construed as a potential conflict of interest.

## Abbreviations

GGT, gamma-glutamyl transpeptidase; GPR, GGT to platelet ratio; ICC, intrahepatic cholangiocarcinoma; INR, international normalized ratio; AUROC, the area under receiver operating characteristic curves; C-index, concordance-index; OS, overall survival; RFS, recurrence-free survival; BCLC staging classification, Barcelona Clinic Liver Cancer staging classification; AJCC TNM system, American Joint Committee on Cancer TNM system; HCC, hepatocellular carcinoma; HBV, hepatitis B virus; HCV, hepatitis C virus; CEA, carcinoembryonic antigen; CA19-9, cancer antigen 19-9; NLR, neutrophil-to-lymphocyte ratio; PLR, platelet-to-lymphocyte ratio; SII, systemic immune-inflammation index; FIB-4, fibrosis-4 score; ALBI, albumin-bilirubin score; MELD, Model for end-stage liver disease; TACE, transarterial chemoembolization; RFA, radiofrequency ablation; ALT, alanine aminotransferase; AST, aspartate aminotransferase; AFP, alpha-fetoprotein; MVI, microvascular invasion; DCA, decision curve analysis; PTCD, percutaneous transhepaticcholangial drainage.
