# Supplementary material for: A Novel Nomogram Based on Hepatic and Coagulation Function for Evaluating Outcomes of Intrahepatic Cholangiocarcinoma After Curative Hepatectomy: A Multi-Center Study of 653 Patients
Source: Front Oncol. 2021 Jul 12;11:711061. doi: 10.3389/fonc.2021.711061 (PMC8311735; doi:10.3389/fonc.2021.711061)
Supplement: Supplementary file 1 [file DataSheet_1.docx]

**cox regression**

library("survival")

library("survminer")

ICC<-read.csv("cumulative hazard versus time.csv",header=T,row.names=1)

res.cox <- coxph(Surv(DFS.months, DFS.status) ~ CA19.9.grade, data =ICC)

test.ph <- cox.zph(res.cox)

test.ph

ggcoxzph(test.ph)

**DCA curves**

library(survival)

library(rms)

library(rmda)

data(package="survival")

ICC<-read.csv("dereviation_nomogram.csv",header=T,row.names=1)

dd<-datadist(ICC)

option<-options(datadist='dd')

f<-cph(Surv(OS.months,OS.status)~Cirrhosis+Hepatolithiasis+Multiple.tumors+poor.tumor.differentiation+LN.metastasis+Perineural.invasion+GPR.INR.score+CA199.22U.ml,data=ICC,x=TRUE,y=TRUE,surv=TRUE)

survival<-Survival(f)

survival1<-function(x)survival(12,x)

survival2<-function(x)survival(36,x)

survival3<-function(x)survival(60,x)

nom<-nomogram(f,fun=list(survival1,survival2,survival3),

fun.at=c(0.05,seq(0.1,0.9,by=0.1),0.95),lp=T,

funlabel=c('1 year survival','3 year survival','5 year survival'))

ICC$linear.predictors<-predict(f,type="lp",data=ICC)

head(ICC)

BCLC<- decision_curve(OS.status~BCLC.stage.B.C.0.A,data= ICC,

family = binomial(link ='logit'),

thresholds= seq(0,1, by = 0.01),

confidence.intervals = 0.95,

study.design = 'cohort')

TNM<- decision_curve(OS.status~TNM.stage.III.IV.I.II,data= ICC,

family = binomial(link ='logit'),

thresholds= seq(0,1, by = 0.01),

confidence.intervals = 0.95,

study.design = 'cohort')

GPR.Nomo<-decision_curve(OS.status~linear.predictors,data= ICC,

family = binomial(link ='logit'),

thresholds= seq(0,1, by = 0.01),

confidence.intervals = 0.95,

study.design = 'cohort')

List<- list(BCLC,TNM,GPR.Nomo)

plot_decision_curve(List,

curve.names=c('BCLC','TNM','GPR.Nomo'),

cost.benefit.axis =FALSE,col= c('brown1','blue','goldenrod2'),

confidence.intervals=FALSE,

standardize = FALSE)

**KM plot**

KMplot<-read.csv("dereviation_cohort.csv",header=T,row.names=1)

library("survival")

library("survminer")

fit <- survfit(Surv(OS.months, OS.status) ~ GPR.grade, data=KMplot)

p3 <- ggsurvplot(fit, data = KMplot,

surv.median.line = "hv",

palette=c("red", "blue"),

legend.labs=c("GPR<0.7","GPR>=0.7"),

legend.title="Group",

title="Overall survival",

ylab="Cumulative survival (percentage)",xlab = " Time (Months)",

censor.shape = 124,censor.size = 2,conf.int = FALSE,

break.x.by = 12,

risk.table = TRUE,tables.height = 0.2,

tables.theme = theme_cleantable(),

ggtheme = theme_bw())

p3

res_cox<-coxph(Surv(OS.months, OS.status) ~GPR.grade, data=KMplot)

p3$plot = p3$plot + ggplot2::annotate("text",x = 50, y = 0.15,

label = paste("HR :",round(summary(res_cox)$conf.int[1],2))) + ggplot2::annotate("text",x = 50, y = 0.10,

label = paste("(","95%CI:",round(summary(res_cox)$conf.int[3],2),"-",round(summary(res_cox)$conf.int[4],2),")",sep = ""))+

ggplot2::annotate("text",x = 50, y = 0.05,

label = paste("P:",round(summary(res_cox)$coef[5],4)))

p3

**nomogram**

library(survival)

library(rms)

data(package="survival")

ICC<-read.csv("nomogram.csv",header=T,row.names=1)

dd<-datadist(ICC)

options(datadist='dd')

f<-cph(Surv(OS.months,OS.status)~Cirrhosis+Hepatolithiasis+Multiple.tumors+poor.tumor.differentiation+LN.metastasis+Perineural.invasion+GPR.INR.score+CA199.22U.ml,data=ICC,x=TRUE,y=TRUE,surv=TRUE)

survival<-Survival(f)

survival1<-function(x)survival(12,x)

survival2<-function(x)survival(36,x)

survival3<-function(x)survival(60,x)

nom<-nomogram(f,fun=list(survival1,survival2,survival3),

fun.at=c(0.05,seq(0.1,0.9,by=0.1),0.95),lp=F,

funlabel=c('1 year survival','3 year survival','5 year survival'))

plot(nom)

f<-coxph(Surv(OS.months,OS.status==1)~Cirrhosis+Hepatolithiasis+Multiple.tumors+poor.tumor.differentiation+LN.metastasis+Perineural.invasion+GPR.INR.score+CA199.22U.ml,data=ICC)

sum.surv<-summary(f)

c_index_se<-sum.surv$concordance

c_index=c_index_se[1]

c_index.ci_low=c_index-c_index_se[2]

c_index.ci_hig=c_index+c_index_se[2]

cal<- calibrate(f,cmethod='KM', method='boot', u=60, m=100, B=100)

plot(cal,lwd=2,lty=1,errbar.col=c(rgb(0,118,192,maxColorValue=255)),xlim=c(0,0.85),ylim=c(0,0.85),xlab="Nomogram-Predicted Probability of 5-Year OS",ylab="Actual 5-Year OS(proportion)", col=c(rgb(192,98,83,maxColorValue=255)))

lines(cal[,c("mean.predicted","KM")],type="b",lwd=2,col=c(rgb(192,98,83,maxColorValue=255)), pch=16)

abline(0,1,lty=3,lwd=2,col=c(rgb(0,118,192,maxColorValue=255)))

**time-dependent AUROC(COX)**

library(survivalROC)

ICC<-read.csv("ICC_cohort_deveriation.csv",header=T,row.names=1)

GPR.grade<-timeROC(T=ICC$OS.months,

delta=ICC$OS.status,marker=ICC$GPR.grade,

cause=1,weighting="cox",

times=c(0,10,20,30,40,50,60))

GPR.grade

INR.grade<-timeROC(T=ICC$OS.months,

delta=ICC$OS.status,marker=ICC$INR.grade,

cause=1,weighting="cox",

times=c(0,10,20,30,40,50,60))

INR.grade

GPR.INR.score<-timeROC(T=ICC$OS.months,

delta=ICC$OS.status,marker=ICC$GPR.INR.score,

cause=1,weighting="cox",

times=c(12,24,36,48,60,72,84,96,108,120))

GPR.INR.score

plotAUCcurve(GPR.grade,conf.int=FALSE,col="red")

plotAUCcurve(INR.grade,conf.int=FALSE,col="blue",add=TRUE)

plotAUCcurve(GPR.INR.score,conf.int=FALSE,col="black",add=TRUE)

legend("topright",c("GPR=0.7","INR=1.1","GPR-INR score"),col=c("red","blue","black"),lty=1,lwd=2)

**timeROC(cox)**

library(timeROC)

library(survival)

ICC<-read.csv("Indexes_grades.csv",header=T,row.names=1)

nobs=NROW(ICC)

ROC.PLR<-timeROC(T=ICC$DFS.months,delta=ICC$DFS.status,

marker=ICC$PLR,cause=1,

weighting="cox"

times=c(12,36,60),ROC=TRUE)

ROC.PLR

ROC.SII<-timeROC(T=ICC$DFS.months,delta=ICC$DFS.status,

marker=ICC$SII,cause=1,

weighting="cox",

times=c(12,36,60),ROC=TRUE)

ROC.SII

ROC.INR<-timeROC(T=ICC$DFS.months,delta=ICC$DFS.status,

marker=ICC$INR,cause=1,

weighting="cox",

times=c(12,36,60),ROC=TRUE)

ROC.INR

ROC.FIB.4<-timeROC(T=ICC$DFS.months,delta=ICC$DFS.status,

marker=ICC$FIB.4,cause=1,

weighting="cox",

times=c(12,36,60),ROC=TRUE)

ROC.FIB.4

ROC.GPR<-timeROC(T=ICC$DFS.months,delta=ICC$DFS.status,

marker=ICC$GPR,cause=1,

weighting="cox",

times=c(12,36,60),ROC=TRUE)

ROC.GPR

ROC.ALBI<-timeROC(T=ICC$DFS.months,delta=ICC$DFS.status,

marker=ICC$ALBI,cause=1,

weighting="cox",

times=c(12,36,60),ROC=TRUE)

ROC.ALBI

ROC.Child.score<-timeROC(T=ICC$DFS.months,delta=ICC$DFS.status,

marker=ICC$Child.score,cause=1,

weighting="cox",

times=c(12,36,60),ROC=TRUE)

ROC.Child.score

plot(ROC.PLR,time=60,col="firebrick")

plot(ROC.SII,time=60,add=TRUE,col="orange")

plot(ROC.INR,time=60,add=TRUE,col="gold")

plot(ROC.FIB.4,time=60,add=TRUE,col="forestgreen")

plot(ROC.GPR,time=60,add=TRUE,col="black")

plot(ROC.ALBI,time=60,add=TRUE,col="darkslateblue")

plot(ROC.Child.score,time=60,add=TRUE,col="darkviolet")

legend("bottomright",cex=0.7,c("GPR:0.593","INR:0.546","PLR:0.573","SII:0.625","FIB-4:0.452","ALBI:0.528"),col=c("black","gold","firebrick","orange","forestgreen","darkslateblue"),lty=1,lwd=2)
