# Supplementary figures and images for: A Novel Nomogram Based on Hepatic and Coagulation Function for Evaluating Outcomes of Intrahepatic Cholangiocarcinoma After Curative Hepatectomy: A Multi-Center Study of 653 Patients
Source: Front Oncol. 2021 Jul 12;11:711061. doi: 10.3389/fonc.2021.711061 (PMC8311735; doi:10.3389/fonc.2021.711061)

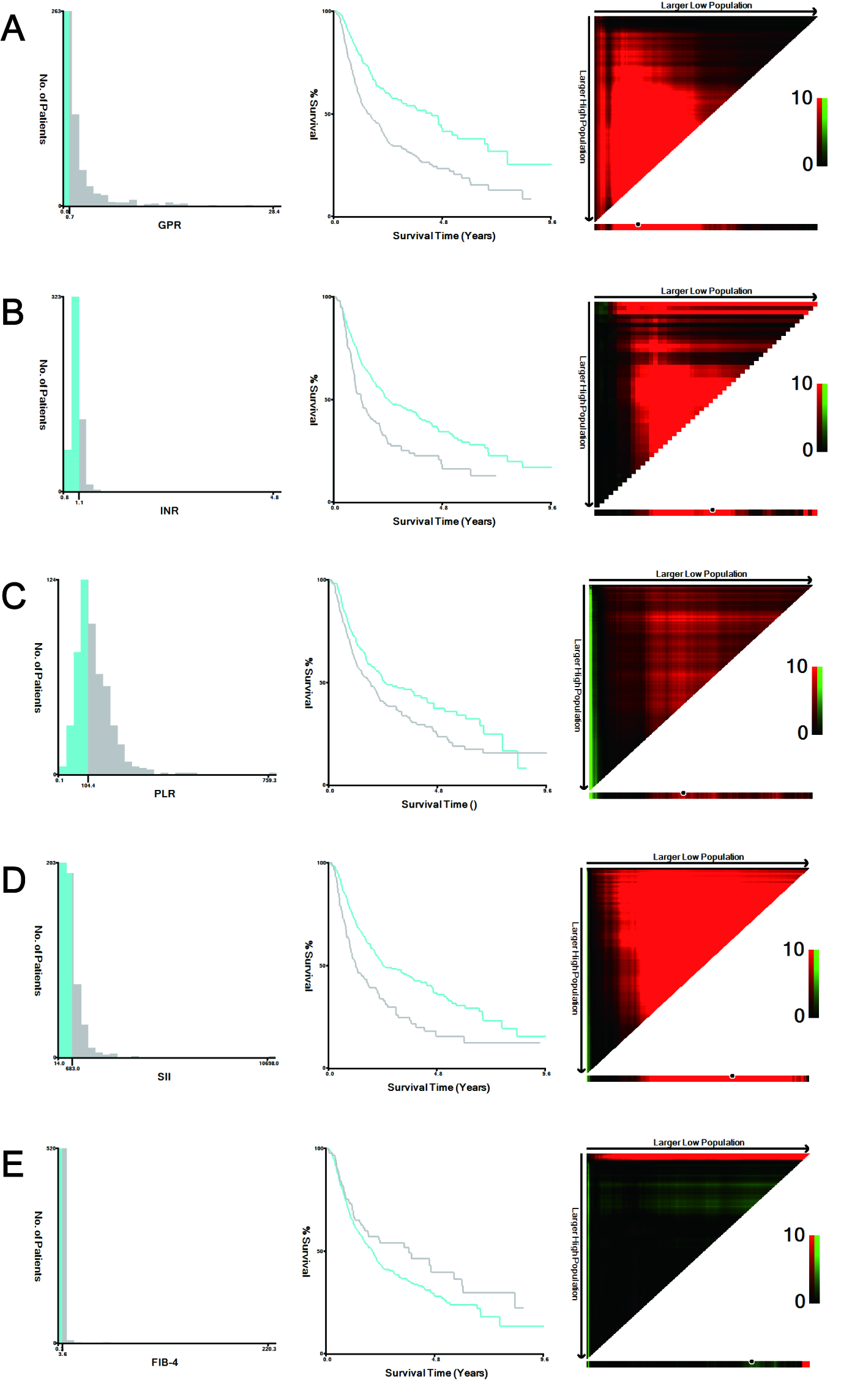

Supplement: Supplementary file 3 [file Image_1.tif]

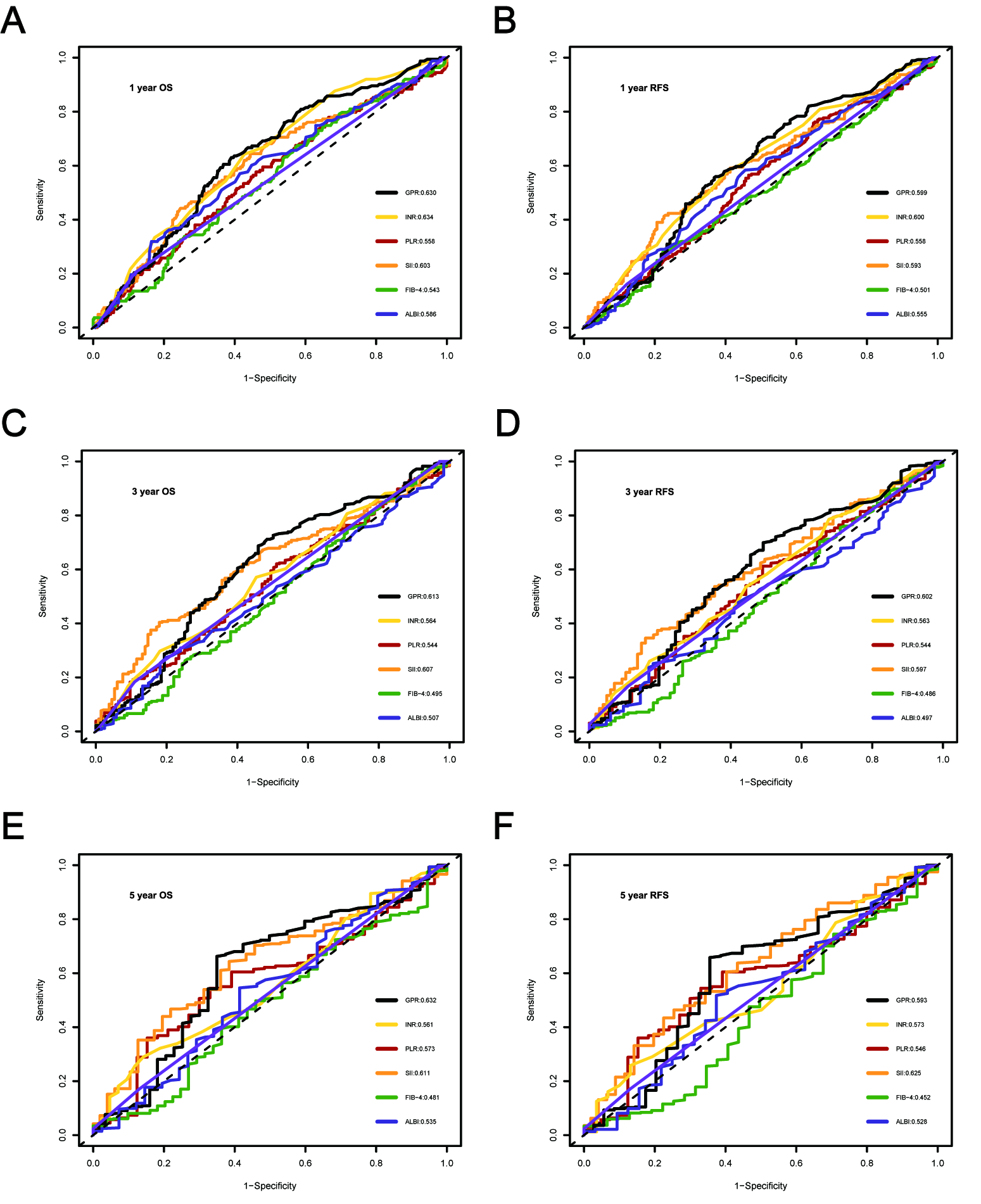

Supplement: Supplementary file 4 [file Image_2.tif]

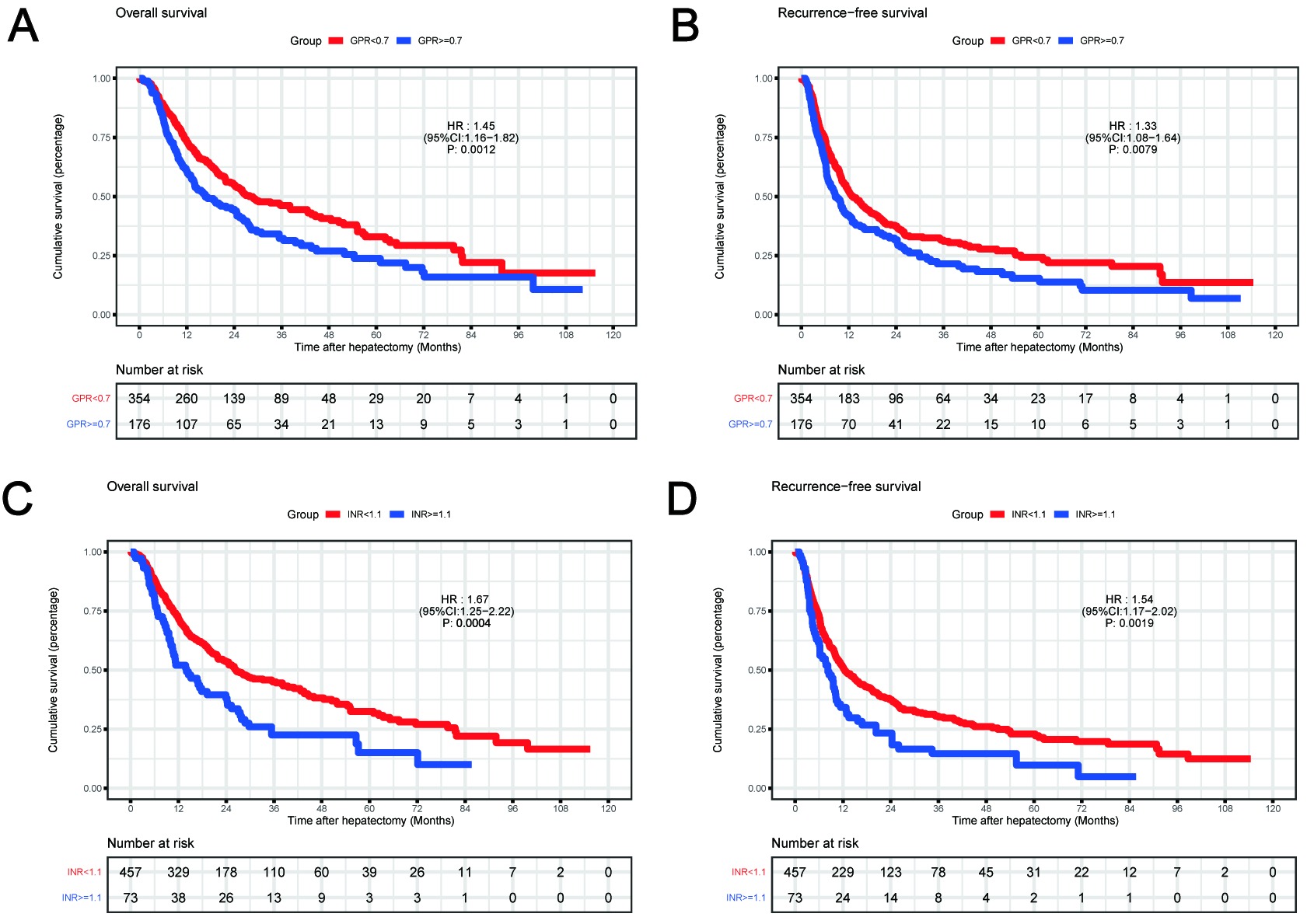

Supplement: Supplementary file 5 [file Image_3.tif]

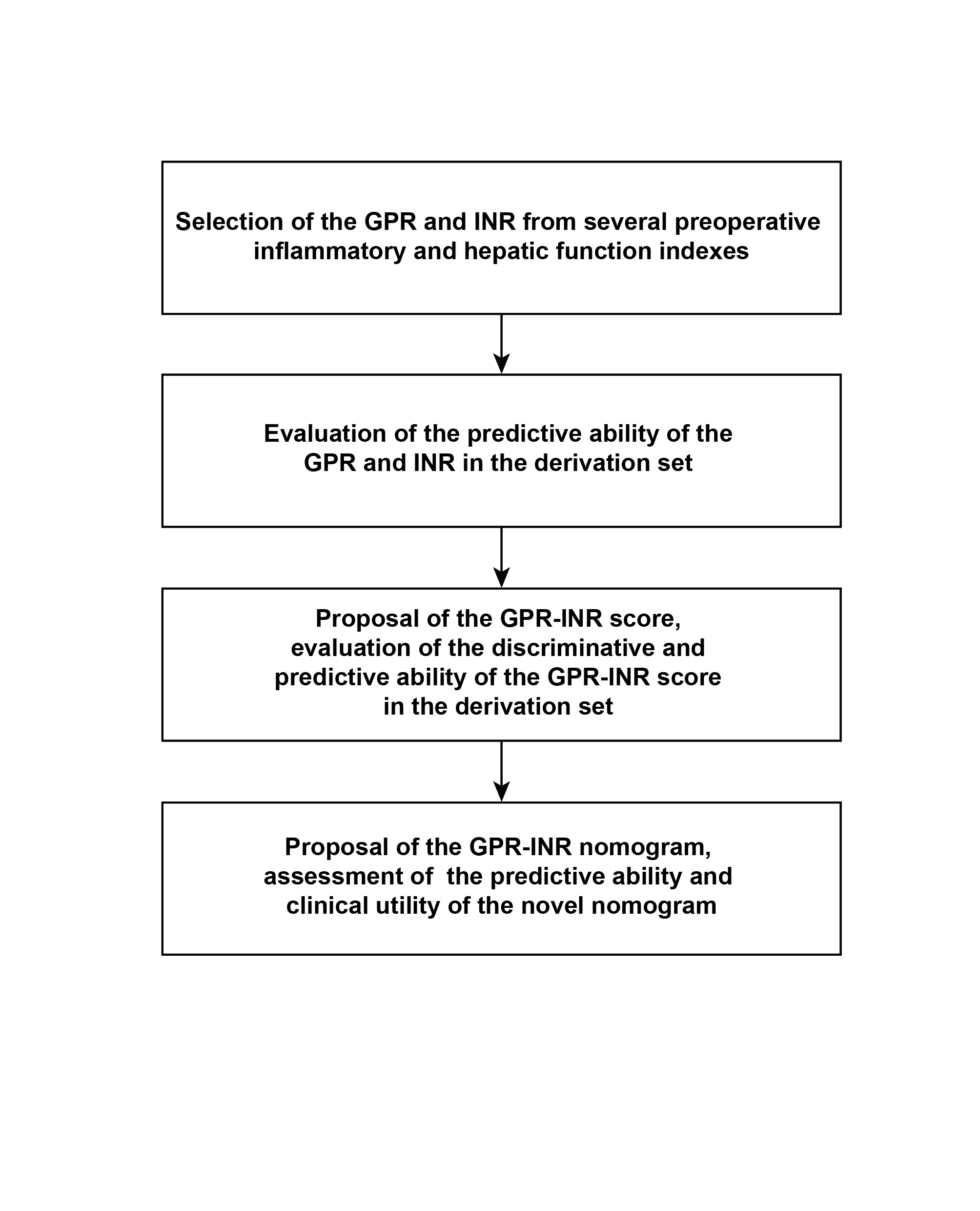

Supplement: Supplementary file 6 [file Image_4.tif]
