# Supplementary material for: A Novel Nomogram Based on Hepatic and Coagulation Function for Evaluating Outcomes of Intrahepatic Cholangiocarcinoma After Curative Hepatectomy: A Multi-Center Study of 653 Patients
Source: Front Oncol. 2021 Jul 12;11:711061. doi: 10.3389/fonc.2021.711061 (PMC8311735; doi:10.3389/fonc.2021.711061)
Supplement: Supplementary file 7 [file Table_1.docx]

**Supplemental tables**

**Supplemental table 1. Correlation of characteristics with the GPR and INR of 530 ICC patients treated with surgical resection in the derivation cohort.**

| **Characteristics** | **GPR** | | | **INR** | | |
| --- | --- | --- | --- | --- | --- | --- |
|  | **<0.7(n=354)** | **≥0.7(n=176)** | **P value** | **<1.1(n=457)** | **≥1.1(n=73)** | **P value** |
| Age, mean ± SD | 57.6±10.7 | 56.6±10.6 | 0.374 | 57.6±10.6 | 54.8±11.1 | 0.034 |
| Gender, n (%) |  |  |  |  |  |  |
| male | 157 (44.4%) | 99 (56.2%) | 0.01 | 209 (45.7%) | 47 (64.4%) | 0.003 |
| female | 197 (55.6%) | 77 (43.8%) |  | 248 (54.3%) | 26 (35.6%) |  |
| Cirrhosis, n (%) | 92 (26.0%) | 56 (31.8%) | 0.159 | 111 (24.3%) | 37 (50.7%) | <0.001 |
| Ascites, n (%) | 30 (8.5%) | 20 (11.4%) | 0.284 | 40 (8.8%) | 10 (13.7%) | 0.179 |
| Multiple tumors, n (%) | 104 (29.4%) | 53 (30.1%) | 0.861 | 134 (29.3%) | 23 (31.5%) | 0.704 |
| Tumor size (cm), mean ± SD | 5.8±2.6 | 6.2±3.0 | 0.127 | 5.8±2.6 | 6.5±3.2 | 0.036 |
| Hepatolithiasis, n (%) | 55 (15.5%) | 33 (18.8%) | 0.349 | 76 (16.6%) | 12 (16.4%) | 0.967 |
| Microvascular invasion, n (%) | 33 (9.3%) | 20 (11.4%) | 0.461 | 46 (10.1%) | 7 (9.6%) | 0.9 |
| Macrovascular invasion, n (%) | 67 (18.9%) | 56 (31.8%) | <0.001 | 103 (22.5%) | 20 (27.4%) | 0.361 |
| Lymph node metastasis, n (%) | 78 (22.0%) | 51 (29.0%) | 0.079 | 106 (23.2%) | 23 (31.5%) | 0.124 |
| Biliary invasion, n (%) | 21 (5.9%) | 32 (18.2%) | <0.001 | 50 (10.9%) | 3 (4.1%) | 0.071 |
| Perineural invasion, n (%) | 44 (12.4%) | 33 (18.8%) | 0.052 | 71 (15.5%) | 6 (8.2%) | 0.099 |
| Liver capsule invasion, n (%) | 234 (66.1%) | 91 (51.7%) | 0.001 | 288 (63.0%) | 37 (50.7%) | 0.044 |
| Poor tumor differentiation, n (%) | 226 (69.5%) | 110 (70.1%) | 0.906 | 287 (69.2%) | 49 (73.1%) | 0.511 |
| CA19-9, n (%) |  |  |  |  |  |  |
| ≥22U/ml | 103 (31.7%) | 35 (22.0%) | 0.027 | 123 (29.5%) | 15 (22.4%) | 0.232 |
| <22U/ml | 222 (68.3%) | 124 (78.0%) |  | 294 (70.5%) | 52 (77.6%) |  |
| HBsAg (positive), n (%) | 113 (32.0%) | 40 (22.9%) | 0.029 | 119 (26.2%) | 34 (46.6%) | <0.001 |
| HCV, n (%) | 2 (0.6%) | 1 (0.6%) | 0.996 | 1 (0.2%) | 2 (2.7%) | 0.008 |
| Child score, n (%) |  |  |  |  |  |  |
| 5 | 313 (88.4%) | 138 (78.4%) | 0.002 | 398 (87.1%) | 53 (72.6%) | 0.001 |
| 6 | 41 (11.6%) | 38 (21.6%) |  | 59 (12.9%) | 20 (27.4%) |  |
| TNM stage, n (%) |  |  |  |  |  |  |
| I-II | 105 (29.7%) | 68 (38.6%) | 0.038 | 145 (31.7%) | 28 (38.4%) | 0.262 |
| III-IV | 249 (70.3%) | 108 (61.4%) |  | 312 (68.3%) | 45 (61.6%) |  |
| BCLC stage, n (%) |  |  |  |  |  |  |
| 0-A | 186 (52.5%) | 82 (46.6%) | 0.197 | 231 (50.5%) | 37 (50.7%) | 0.983 |
| B-C | 168 (47.5%) | 94 (53.4%) |  | 226 (49.5%) | 36 (49.3%) |  |
| Overall survival, month, mean ± SD | 25.8±21.2 | 23.1±21.6 | 0.027 | 25.4 ± 21.6 | 21.3 ± 19.5 | 0.048 |

Abbreviations: CA19-9, cancer antigen 19-9; HBsAg, hepatitis B surface antigen; HCV, hepatitis C virus; BCLC stage, Barcelona Clinic Liver Cancer stage; INR, international normalized ratio; GPR, gamma-glutamyl transpeptidase to platelet ratio.

**Supplemental table 2. Correlation of characteristics with the GPR and INR of 530 ICC patients treated with surgical resection in the derivation cohort.**

| **Characteristics** | **GPR** | | | **INR** | | |
| --- | --- | --- | --- | --- | --- | --- |
|  | **<0.7(n=55)** | **≥0.7(n=68)** | **P value** | **<1.1(n=80)** | **≥1.1(n=43)** | **P value** |
| Age, mean ± SD | 58.3 ± 11.5 | 58.1 ± 10.9 | 0.907 | 58.6 ± 11.0 | 57.4 ± 11.5 | 0.64 |
| Gender, n (%) |  |  |  |  |  |  |
| male | 18 (32.7%) | 34 (50.0%) | 0.054 | 47 (58.8%) | 24 (55.8%) | 0.753 |
| female | 37 (67.3%) | 34 (50.0%) |  | 33 (41.2%) | 19 (44.2%) |  |
| Cirrhosis, n (%) | 6 (10.9%) | 10 (14.7%) | 0.599 | 8 (10.0%) | 8 (18.6%) | 0.26 |
| Ascites, n (%) | 26 (47.3%) | 34 (50.0%) | 0.764 | 36 (45.0%) | 24 (55.8%) | 0.253 |
| Multiple tumors, n (%) | 12 (21.8%) | 24 (35.3%) | 0.102 | 21 (26.2%) | 15 (34.9%) | 0.316 |
| Tumor size (cm), mean ± SD | 5.1±3.4 | 5.7±2.8 | 0.137 | 5.4±2.3 | 5.9±3.4 | 0.182 |
| Hepatolithiasis, n (%) | 5 (9.1%) | 18 (26.5%) | 0.014 | 11 (13.8%) | 12 (27.9%) | 0.088 |
| Microvascular invasion, n (%) | 12 (21.8%) | 17 (25.0%) | 0.679 | 15 (18.8%) | 14 (32.6%) | 0.085 |
| Macrovascular invasion, n (%) | 17 (30.9%) | 8 (11.8%) | 0.009 | 14 (17.5%) | 11 (25.6%) | 0.349 |
| Lymph node metastasis, n (%) | 6 (10.9%) | 29 (42.6%) | <0.001 | 17 (21.2%) | 18 (41.9%) | 0.016 |
| Biliary invasion, n (%) | 27 (49.1%) | 36 (52.9%) | 0.671 | 40 (50.0%) | 23 (53.5%) | 0.712 |
| Perineural invasion, n (%) | 8 (14.5%) | 21 (30.9%) | 0.034 | 17 (21.2%) | 12 (27.9%) | 0.407 |
| Liver capsule invasion | 25 (45.5%) | 40 (58.8%) | 0.14 | 42 (52.5%) | 23 (53.5%) | 0.917 |
| Poor tumor differentiation, n (%) | 37 (67.3%) | 53 (77.9%) | 0.184 | 56 (70.0%) | 34 (79.1%) | 0.279 |
| CA19-9, n (%) |  |  |  |  |  |  |
| ≥22U/ml | 21 (38.2%) | 9 (13.2%) | 0.001 | 23 (28.7%) | 7 (16.3%) | 0.125 |
| <22U/ml | 34 (61.8%) | 59 (86.8%) |  | 57 (71.2%) | 36 (83.7%) |  |
| HBsAg (positive), n (%) | 10 (18.2%) | 12 (17.6%) | 1 | 13 (16.2%) | 9 (20.9%) | 0.623 |
| HCV, n (%) | 1 (1.8%) | 0 (0.0%) | 0.447 | 1 (1.2%) | 0 (0.0%) | 1 |
| Child score, n (%) |  |  |  |  |  |  |
| 5 | 46 (83.6%) | 47 (69.1%) | 0.062 | 73 (91.2%) | 20 (46.5%) | <0.001 |
| 6 | 9 (16.4%) | 21 (30.9%) |  | 7 (8.8%) | 23 (53.5%) |  |
| TNM stage, n (%) |  |  |  |  |  |  |
| I-II | 36 (65.5%) | 50 (73.5%) | 0.332 | 58 (72.5%) | 28 (65.1%) | 0.395 |
| III-IV | 19 (34.5%) | 18 (26.5%) |  | 22 (27.5%) | 15 (34.9%) |  |
| BCLC stage |  |  |  |  |  |  |
| 0-A | 37 (67.3%) | 48 (70.6%) | 0.692 | 54 (67.5%) | 31 (72.1%) | 0.599 |
| B-C | 18 (32.7%) | 20 (29.4%) |  | 26 (32.5%) | 12 (27.9%) |  |
| Overall survival, month, mean ± SD | 55.8 ± 27.7 | 20.4 ± 20.8 |  | 39.6 ± 30.6 | 29.8 ± 27.5 |  |

Abbreviations: CA19-9, cancer antigen 19-9; HBsAg, hepatitis B surface antigen; HCV, hepatitis C virus; BCLC stage, Barcelona Clinic Liver Cancer stage; INR, international normalized ratio; GPR, gamma-glutamyl transpeptidase to platelet ratio.

**Supplemental figure legends**

**Supplemental figure 1.** Identification for the cutoff points produced by X-tile plot in the derivation set. The prognostic power was strongest when the cutoff value of GPR was 0.7 (A); INR, 1.1 (B); PLR, 104.4 (C); SII, 683 (D); FIB-4, 3.6 (E).

**Supplemental figure 2.** The ROC curves analysis to compare the ability of GPR, INR, PLR, SII, FIB-4 and ALBI in predicting 1-year OS (A), RFS (B); 3-year OS (C), RFS (D); 5-year OS (E), RFS (F) in derivation set.

**Supplemental figure 3.** The Kaplan–Meier curves of the patients in the derivation set for OS and RFS, patients with GPR≥0.7 had worse OS (A) and RFS (B) than patients with GPR<0.7; patients with INR≥1.1 had worse OS (C) and RFS (D) than patients with INR<1.1.
